# Triple spondylolyse bilatérale lombaire étagée

**DOI:** 10.11604/pamj.2017.27.105.12235

**Published:** 2017-06-12

**Authors:** Noukhoum Koné, Outouma Soumaré

**Affiliations:** 1 Service de Neurochirurgie, Centre Hospitalier de Kiffa, Mauritanie; 2 Service de Neurochirurgie, Centre Hospitalier des Spécialités, Nouakchott, Mauritanie

**Keywords:** Lombalgie, spondylolyse lombaire étagée, traitement conservateur, Low back pain, multilevel lumbar spondylolysis, conservative treatment

## Image en médecine

Nous rapportons le cas d'une patiente de 30 ans, sans antécédents pathologiques particuliers, qui présente depuis 6 mois des lombalgies sans notion de radiculalgie aux membres inférieurs. L'examen neurologique retrouve un syndrome rachidien lombaire sans déficit neurologique associé. Le scanner du rachis lombaire en coupes sagittales paramédianes droite (A) et gauche (B) montre une triple spondylolyse bilatérale L2, L3, L4 associée à un spondylolisthésis L3-L4 de grade I de Meyerding. L'évolution clinique est marquée par une disparition de la symptomatologie par le traitement médical à base d'antalgique, d'anti-inflammatoire non stéroïdien et de ceinture de stabilisation lombaire au bout de deux semaines de traitement. L'incidence des spondylolyses lombaires dans la population adulte est d'environ 6% et concernent dans plus de 90% des cas la vertèbre L5. Les spondylolyses lombaires étagées sont inhabituelles, elles représentent 1.2% à 5.6% des spondylolyses lombaires et il s'agit dans plus de 60% des cas d'une atteinte de 2 niveaux, L4 et L5. Le traitement conservateur avec un suivi régulier est indiqué en cas d'absence de symptômes neurologiques avec ou sans spondylolisthésis inférieur à 25%.

**Figure 1:**
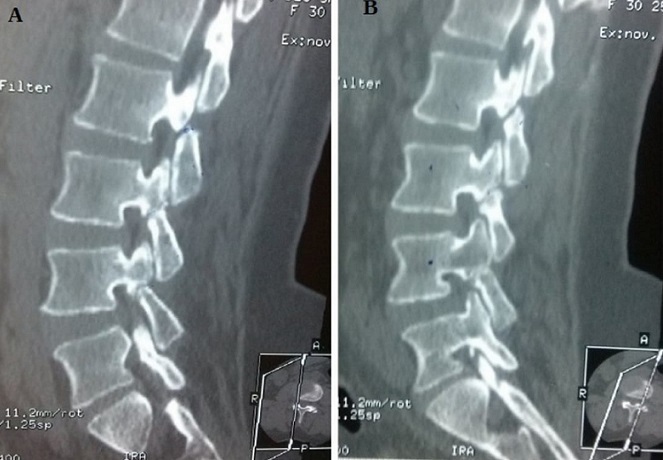
Coupes sagittales paramédianes droite (A) et gauche (B) d'une TDM lombo-sacrée montrant une lyse isthmique étagée L2, L3,L4 bilatérale associée à un spondylolisthésis L4-L5

